# Carbon Nanoparticle Effects on PAN Crystallization for Higher-Performance Composite Fibers

**DOI:** 10.1021/acspolymersau.5c00006

**Published:** 2025-04-09

**Authors:** Xiao Sun, Xiaoli Li, Varunkumar Thippanna, Conor Doyle, Ying Mu, Thomas Barrett, Lindsay B. Chambers, Churan Yu, Yiannis Levendis, Kenan Song, Marilyn Minus

**Affiliations:** † Department of Mechanical and Industrial Engineering, Northeastern University, 360 Huntington Avenue, Boston 02115, Massachusetts, United States; ‡ Mechanical Engineering, College of Engineering, 1355 University of Georgia, 302 E Campus Rd, Athens 30602, Georgia, United States; § Mechanical Engineering, College of Engineering, University of Georgia (UGA), 302 E. Campus Rd., Athens 30602, Georgia, United States

**Keywords:** polyacrylonitrile fibers, wet spinning, carbon nanotubes, composite fibers, crystallization

## Abstract

Polyacrylonitrile (PAN) fibers, widely recognized for their exceptional carbonization and graphitization at higher processing temperatures, serve as precursors for high-performance carbon fiber production. This study explores the fabrication of PAN control fibers and PAN-CNT composites via fiber spinning, a process influenced by solution behavior, macromolecular extension, and crystallizations. The polymer chain morphologies, along with pore nucleation and growth, play a critical role in determining fiber microstructure and mechanical properties. Comprehensive characterization like wide-angle X-ray diffraction (WAXD) and differential scanning calorimetry (DSC) was conducted for PAN control and PAN-CNT composite fibers at polymer concentrations of 9, 10, and 11 wt % with specific CNT loading. This study highlights the enhanced performance of PAN fibers and PAN/CNT composite fibers fabricated at polymer concentrations of 9, 10, and 11 wt %. Additionally, the effects of carbon nanotubes (CNTs) on the polymer microstructure and properties, including crystallinity and thermal stability, were analyzed and compared.

## Graphical abstract

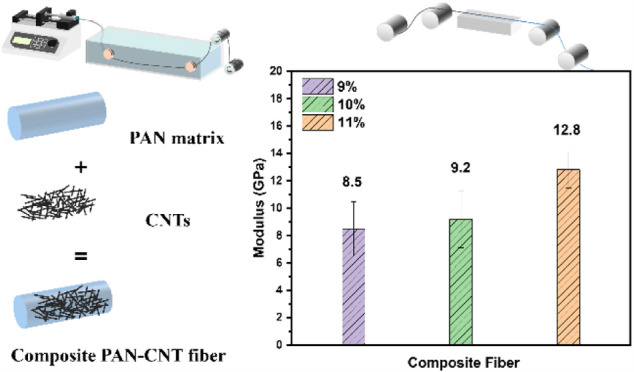

## 1 Introduction

Polyacrylonitrile (PAN) fibers play a critical role in high-performance materials, not only as a key player in the textile industry but also as the primary precursor for carbon fiber production.^1,2^ Carbon fibers, known for their exceptional mechanical and thermal properties, are widely used in industries such as aerospace, automotive, and construction.^3,4^ The performance of carbon fibers is directly linked to the quality of their precursor PAN fibers.^5^ Achieving high-strength and high-modulus PAN fibers requires precise control of molecular alignment, crystallinity, and structural integrity, which ultimately translate into improved properties of the resulting carbon fibers.^6^ Therefore, the development of PAN fibers with optimized structural properties is a key objective for advancing carbon fiber technology.^7,8^

Among the many factors influencing PAN fiber properties, the concentration of PAN in the spinning dope plays a pivotal role in determining spinning dynamics and the resulting fiber characteristics.^9^ Changes in PAN concentration significantly affect molecular interactions within the polymer matrix, leading to variations in the mechanical performance of the fibers. At low concentrations, the polymer matrix may not form a well-integrated network, leading to weaker mechanical properties due to insufficient inter- or intramolecular interactions.^10^ Conversely, high concentrations may lead to elevated chain entanglement that pose challenges during fiber spinning and postprocessing, in addition to other defects, such as chain ends and misalignment. Besides, excessively high concentrations can result in increased solution viscosity, potentially delaying phase separation and affecting the fiber’s structural uniformity. Proper PAN concentrations will enhance fiber formation, tensile strength, and intermolecular bonding while promoting optimal crystallization behavior, leading to improved alignment and packing of polymer chains. Understanding the relationship between PAN concentration and fiber properties is essential for optimizing the spinning process.

One promising approach to further enhance PAN fiber properties is the incorporation of nanoparticles, among which carbon nanotubes (CNTs) are particularly effective. CNTs possess remarkable intrinsic properties, including high tensile strength, lightweight characteristics, excellent thermal conductivity, and a high aspect ratio, making them highly efficient reinforcements for polymer fibers.^11–14^ CNTs not only improve the mechanical and thermal properties of PAN fibers but also influence their crystallization behavior.^15–20^ By interacting with the polymer chains, CNTs facilitate molecular alignment and enhance stress transfer between the matrix and the nanofillers.^21,22^ However, achieving uniform dispersion of CNTs within the polymer matrix remains a significant challenge due to their hydrophobic nature and large surface area.^23^ Despite these challenges, CNT-reinforced PAN fibers have shown promising results, with applications in high-performance fields such as aerospace and automotive.^24^

This study examines the combined effects of PAN concentration and CNT incorporation on the morphology, crystallinity, and mechanical properties of PAN fibers. By systematically adjusting PAN concentrations and embedding CNTs into the polymer matrix, the research explores how these factors influence polymer chain microstructure and their impact on fiber performance, including mechanical and thermal properties. The findings offer valuable insights into optimizing processing parameters to enhance mechanical and thermal robustness, contributing to the development of high-performance PAN fibers and their carbon fiber derivatives.

## 2 Materials and Methods

### 2.1 Materials

The poly­(acrylonitrile-*co*-methacrylic acid) (PAN) used here is a random copolymer with a methacrylic acid content of 0.5 wt % and a molecular weight of approximately 230 kg/mol/a density of 1.18 g/cm^3^, from Goodfellow Co. The solvent used in this study is dimethylformamide (DMF), from Fisher Chemical (certified ACS, 99.9%) and used without further purification. The mixed CNTs used in this study consisted of a combination of single-walled and double-walled carbon nanotubes (MWNTs) obtained from Cheaptubes Co. (i.e., MWNT content >5 wt % with an outer diameter 1–4 nm, an inner diameter of 0.8–1.6 nm, and a length of 5–30 μm, as well as a density of 1.8 g/cm^3^). Deionized water (DI) is employed as nonsolvent.

### 2.2 Characterization

A Hitachi S-4800 scanning electron microscope (SEM) was used to observe the surfaces of the fibers. Wide-angle X-ray diffraction (WAXD) patterns were obtained using a Rigaku RAPID II equipped with a curved detector from Rigaku Americas Corp.^24^ The operating conditions included a tube voltage of 40 kV and a tube current of 30 mA, using Cu Kα radiation with a wavelength of 0.1541 nm. Data conversion and analysis were performed using Jade software. Static tensile tests were conducted via a dynamic mechanical analyzer (DMA) tester with a loading gap of 15 mm (RSA-G2, TA Instruments), and data from these tests were collected with TRIOS software (TA Instruments).^25–27^ Differential scanning calorimetry (DSC) was carried out on various paste film samples using a DSC25 (TA Instruments) with a T-Zero pan and lid. Samples were equilibrated at 25 °C, then ramped to 400 °C at a rate of 10 °C min^–1^. To determine the fiber weight percentage, thermogravimetric analysis (TGA) was performed using an simultaneous thermal analysis (SDT) 650 system (TA Instruments), heating the samples to 1000 °C at 10 °C/min under a nitrogen atmosphere.

### 2.3 Dope Preparation (Control PAN Fibers)

For the control PAN fibers, 2.8, 3.15, and 3.5 g of PAN powders were separately dissolved in 30 mL of DMF solvent at targeted dissolution temperatures (i.e., a solution temperature of 90 °C) to form 9, 10, and 11 wt % PAN-DMF spinning dopes, respectively. To ensure complete dissolution, the solution was stirred using an overhead mechanical stirrer at 350 rpm for 1 h. The solution was then poured into a 30 mL syringe and placed in a 90 °C vacuum oven for 5 min to remove any potential bubbles. The details of the calculations for different polymer concentrations are provided in the Supporting Information (SI).

### 2.4 Dope Preparation (PAN-CNTs Composite Fibers)

First, 55 mg of PAN powder was dissolved in 220 mL of DMF at 90 °C. An equal amount of 55 mg CNTs was then dispersed into the polymer solution and sonicated for 24 h using a bath sonicator (Fisher FS30, frequency 43 kHz, power 150 W). The same amounts of PAN and CNTs were chosen because strong interfacial interaction between the polymer and CNTs is critical for achieving high mechanical reinforcement and performance. Following sonication, the PAN-CNTs dispersion underwent solution crystallization by shearing the dispersion at 90 °C at a constant speed of 350 rpm.^23^ Vacuum distillation was then employed to remove half the solvent volume under controlled time and temperature conditions. Subsequently, the system was cooled to 60 °C, and nonsolvent water (also at 60 °C) was added to the dispersion at a solvent/nonsolvent ratio of 1/2, followed by stirring for 24 h on a hot plate. After equilibrating at 60 °C, the system was cooled to room temperature and filtered through a 0.45 μm nylon membrane (Millipore) under controlled vacuum. The filtration process is shown in Figure S1. The ink-like paste obtained from filtration was then added to 9, 10, and 11 wt % PAN-DMF solutions to form PAN-CNT composite dopes. The average CNT content in the paste solution after filtration is 51.75 mg. For a polymer concentration of 9 wt %, the CNT content is 1.8 wt %; for 10 wt %, it is 1.6 wt %; and for 11 wt %, it is 1.5 wt %. The details for calculating the CNT-to-polymer ratio are provided in the SI. To ensure complete dissolution, the mixture was stirred with an overhead mechanical stirrer at 350 rpm for 1 h. This process produced composite fibers with varying PAN and CNT ratios.^23,28^

### 2.5 Dynamic Mechanical Analysis (DMA) Test (11 wt % PAN-CNTs Composite Fibers)

Dynamic mechanical analysis (DMA) was performed on an RSA-GA to evaluate the stability of PAN-CNT (11 wt %) fibers under water immersion conditions. The experiments were conducted at 1 Hz with a heating rate of 3 °C/min, ranging from 25 to 100 °C, on ten fiber bundle samples using a gauge length of 15 mm.

### 2.6 Rheological Measurements of 5, 9, 10, 11, and 15 wt % PAN/DMF Solutions

Rheological measurements were performed on a Discover Hybrid Rheometer HR2 (TA Instruments) using a cone-and-plate geometry. A 2 mL sample was placed on a 40 mm, 2° Peltier cone steel plate. Viscosity values were recorded over a shear rate range of 0.01–1000 s^–1^ at both 25 and 50 °C, with a 100 μm truncation gap and a 50 μm trim gap offset. To prevent edge fracture, the sample was slightly overfilled, and any excess solution was carefully removed before testing. Each sample was measured twice to minimize systematic errors.

## 3 Results and Discussion

### 3.1 Overview

Fiber production techniques such as dry spinning, wet spinning, dry-jet wet spinning, and electrospinning are capable of producing ultrafine fibers with exceptional mechanical properties.^29–37^ Among these, wet spinning is the most widely used technique due to its versatility in tailoring the structural, mechanical, and thermal properties of fibers.^38–46^ However, the production of PAN fibers through wet spinning is a complex process influenced by phase separation, rheology, and diffusion phenomena.^47,48^ Upon entering the coagulation bath, the polymer solution undergoes two diffusional movements: solvent diffuses out of the fiber into the bath, while the coagulation bath solution diffuses into the fiber.^49,50^ When the concentrations of polymer, solvent, and coagulant surpass phase equilibrium conditions, the polymer precipitates into fiber form.^51^ The kinetics of this phase separation play a pivotal role in determining the fiber’s final structure and properties.^52^ Figure 1 illustrates our setup for the formation of PAN-CNTs composite fibers. Figure 1a–f show the dope preparation process, which includes the dissolution of PAN in DMF, dispersion and sonication of CNTs, induction of nonsolvent (DI water), and vacuum filtration to collect the wet paste blend. These steps are described in detail in Section 2. In Figure 1g, the wet-spinning process is depicted, where the coagulation solvent (methanol) is pumped into plastic tubing (3 mm inner diameter) and maintained at room temperature. A syringe pump introduces the PAN-CNT dope solution into the methanol-filled tubing through a 20 mL stainless steel syringe connected to a 22-gauge Kel-F Hub Needle (Hamilton Co., catalog number 7750-13). The dope injection rate is maintained at 0.3 mL min^–1^, while the methanol flow rate in the coagulation bath ensures consistent fiber formation. Upon introduction into the flowing methanol, as-spun fibers form immediately and are collected on take-up rollers at a speed of approximately 5 m/min. These processing parameters, such as the methanol flow rate, injection rate of the PAN-CNT dope solution, and take-up roller speed, are carefully optimized to ensure consistent fiber formation, uniform coagulation, and controlled microstructure development during the wet-spinning process. In Figure 1h, the as-spun fibers are coagulated further in methanol for 36 h and then undergo postprocessing through heat-drawing on a hot plate (10 in. by 1 in.).

**Figure 1:**
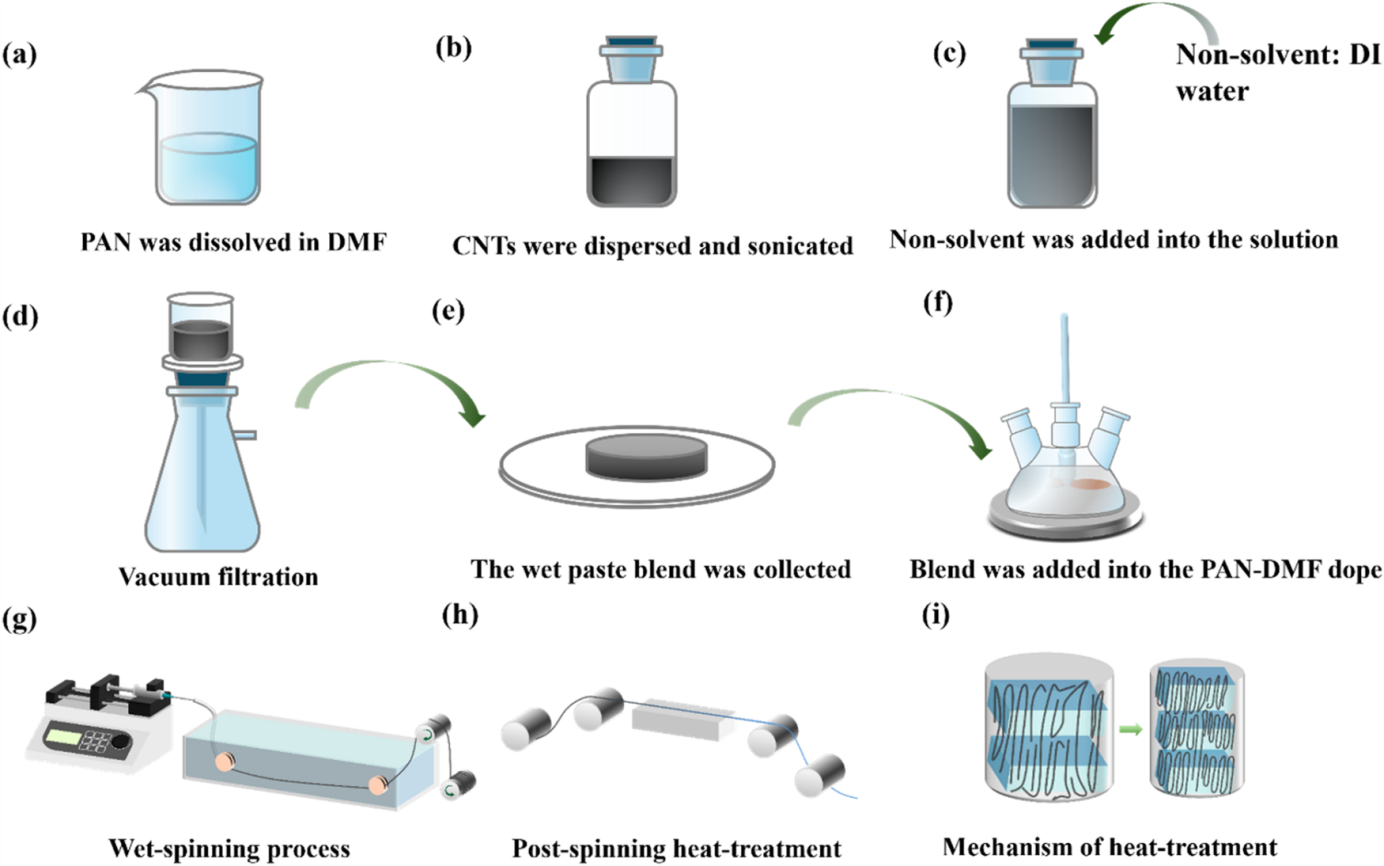
Schematic illustration of the setup for PAN-CNT composite fiber formation: (a–f) Dope preparation process, including PAN dissolution in DMF, CNT dispersion and sonication, nonsolvent addition (DI water), vacuum filtration, wet paste blend collection, and blending into the PAN-DMF dope with Figure S1 in the SI showing the vacuum filtration and paste collection procedure. (g) Wet-spinning process of PAN-CNT composite fibers. (h) Postspinning heat treatment of fibers. (i) Hot-drawing mechanism of fibers during different stages to align molecular structures and enhance properties.

Figure 1i illustrates the mechanism of hot drawing, where the process aligns molecular chains within the fibers at various drawing stages. This alignment improves the fibers’ tenacity, decreases porosity, and enhances compactness, resulting in significantly improved mechanical properties. The process demonstrates the integration of molecular alignment and structure control in producing high-performance PAN-CNT composite fibers.

### 3.2 Concentration Effects on PAN Fibers

Our study focuses on 9–11 wt % PAN concentrations, as this range effectively balances spinnability, polymer crystallization, and fiber mechanical performance while maintaining process feasibility in wet spinning. Spinnability of PAN solutions is critical for fiber manufacturing, and rheological measurements of spinning solutions are often used to guide polymer fiber spinning. The rheological measurement results are presented in Figure S2. Figure S2a, conducted at 25 °C, shows that the 5 wt % polymer concentration exhibits the lowest viscosity. As the PAN content increases, the polymer solutions display higher viscosities, and the transition to shear-thinning behavior occurs at progressively lower shear rates. At a shear rate of approximately 10 s^–1^, the viscosities for the PAN/DMF solutions are 2.9 Pa·s at 5 wt %, 22.9 Pa·s at 9 wt %, 26.6 Pa·s at 10 wt %, 32.2 Pa·s at 11 wt %, and 183.6 Pa·s at 15 wt %. Figure S2b presents results obtained at 50 °C, a temperature employed during fiber spinning by using a heat pad to further improve spinnability. Under these conditions, the 5 wt % solution exhibits the smallest viscosity of 1.5 Pa·s at a shear rate of approximately 10 s^–1^. Under the same conditions, the 9 wt % solution has a viscosity of 15.4 Pa·s, and the 11 wt % solution shows 17.2 Pa·s. Comparing these measurements with those obtained at 25 °C reveals that viscosity decreases at higher temperatures for the same polymer concentration. Excessively high PAN concentrations can hinder optimal crystallization and molecular alignment, negatively impacting mechanical properties. Conversely, lower PAN concentrations (below 9 wt %) may lead to insufficient polymer chain entanglement, resulting in weaker fiber structures and lower mechanical performance, making them less suitable for high-strength applications. While lower concentrations could be explored for lightweight or flexible fiber applications, they would likely require additional modifications to enhance mechanical integrity. Also based on rheological measurement results, our selection of 9–11 wt % PAN represents an optimized balance between processability and mechanical performance for precursor fibers, ensuring smooth fiber formation, controlled crystallization, and optimal mechanical properties.

Figure 2 showcases the effect of varying PAN concentrations on the mechanical properties of the resulting fibers. As the PAN concentration increases, both modulus and strength improve, highlighting a positive correlation between concentration and mechanical performance. The highest values were achieved at 11 wt % PAN, with a modulus average of 8.3 GPa and a strength average of 392 MPa. Conversely, the 9 wt % PAN solution exhibited the lowest mechanical properties, with a modulus average of 5.9 GPa and a strength average of 285 MPa. At 10 wt %, the modulus and strength average values were 6.8 GPa and 318 MPa, respectively, serving as intermediate values. These mechanical properties were tested on the highest-drawn fibers expected with the maximum polymer chain extension and optimized crystallization. Concentrations exceeding 11 wt % were not investigated due to the high viscosity of the dope, which hindered its extrusion into the coagulation bath for fiber formation. This is also consistent with our previous studies and reports.^7,16,23^ Additionally, we conducted a comparative test on 9 wt % PAN-CNT fibers after hot drawing, using the traditional method without DI water and our unique method incorporating DI water, as shown in Figure S3. From the photograph, we can clearly observe black particle spots appearing in fibers processed without DI water, whereas fibers processed with DI water appear darker and have a smoother surface. Furthermore, mechanical analysis comparing the modulus and strength of fibers processed with and without DI water demonstrates that the unique method incorporating DI water results in higher fiber quality.

**Figure 2:**
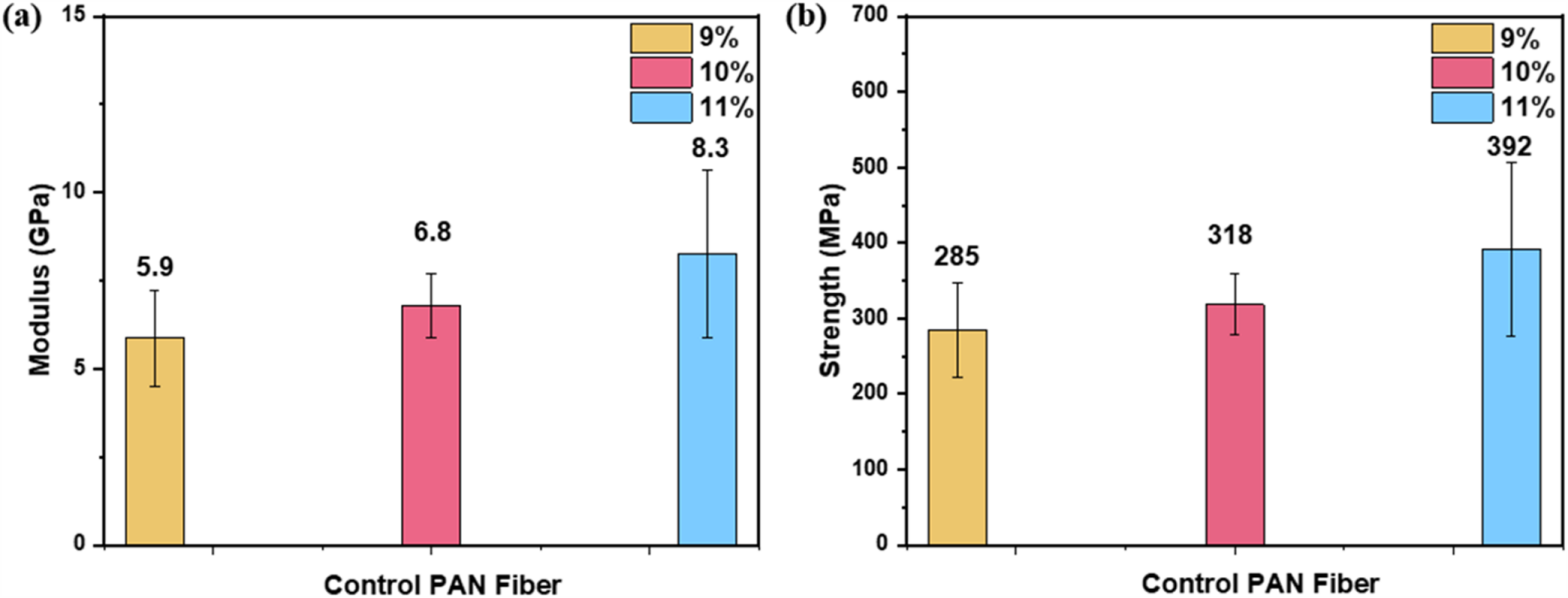
(a) Modulus and (b) strength of PAN control fiber from different PAN/DMF concentrations of 9, 10, and 11 wt %.

Figure 3 presents SEM images of the surface morphology of PAN control fibers prepared at different polymer concentrations of 9 wt % (Figure 3a), 10 wt % (Figure 3b), and 11 wt % (Figure 3c) under magnifications of 2k, 5k, and 20k. At 9 wt %, the fibers exhibit a rough surface with noticeable striations and grooves aligned along the fiber axis, indicating incomplete alignment or structural uniformity. With an increase in concentration to 10 and 11 wt %, the fiber surfaces become progressively smoother, and the surface striations diminish significantly, suggesting improved uniformity and alignment at higher concentrations. The higher polymer concentrations in the solvent facilitate better polymer chain entanglement and interactions during the spinning process, which leads to enhanced chain alignment along the fiber axis. As a result, this alignment reduces surface irregularities, resulting in smoother fiber surfaces. Moreover, higher polymer concentrations promote more uniform packing and stretching of polymer chains during fiber formation, which can positively impact polymer crystallinity and crystallization behavior and will be examined in the following section. The reduction in surface defects and increased alignment at higher concentrations (Figure 3) also suggest improved molecular ordering, which is a critical factor for achieving better mechanical properties and enhanced structural integrity in the fibers. This improved crystallinity is essential for applications requiring high-performance fibers with consistent and predictable properties.^26,53–56^

**Figure 3:**
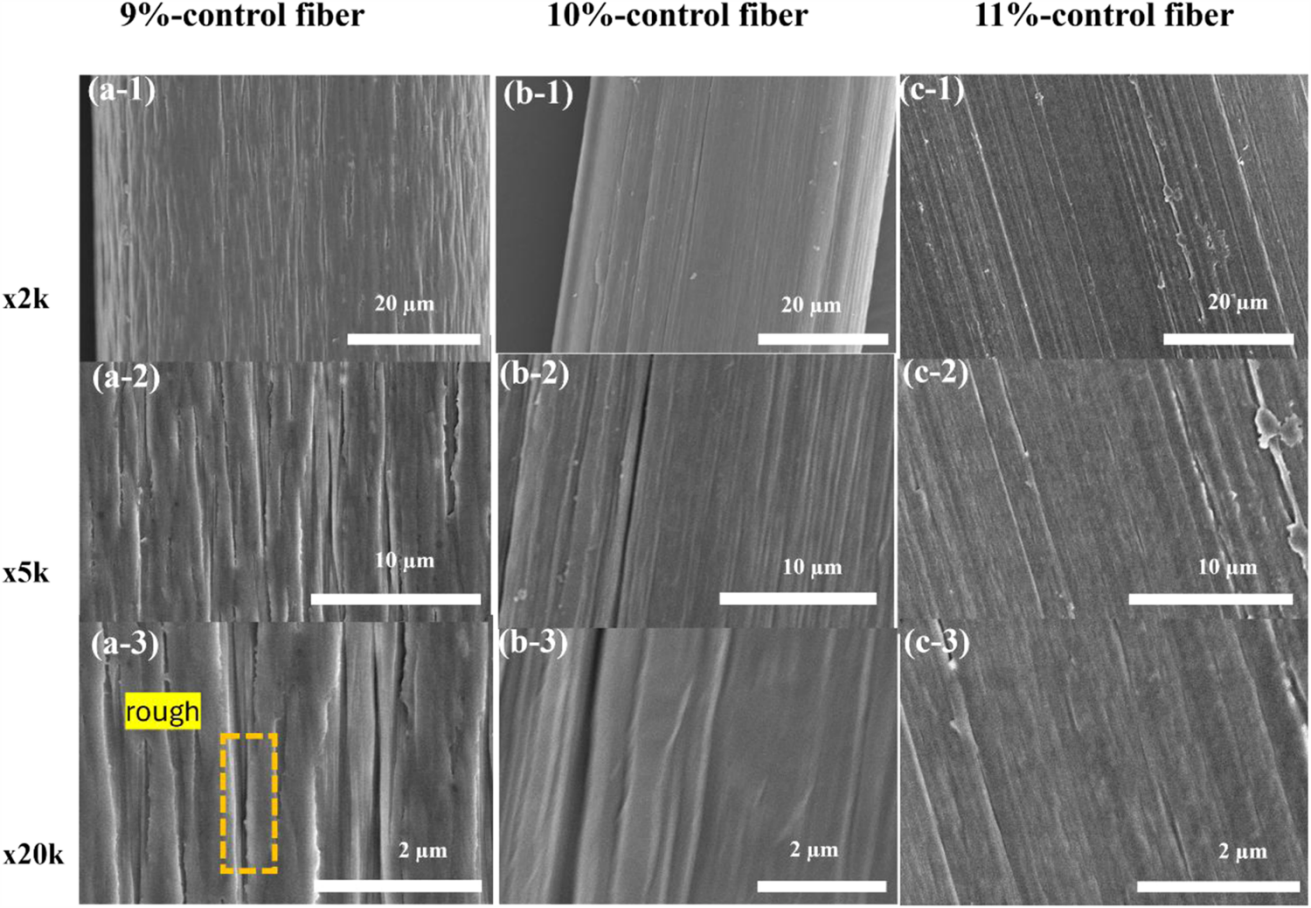
SEM images of PAN control fiber surfaces prepared from different PAN/DMF concentrations under varying magnifications: (a) 9 wt % PAN control fibers, (b) 10 wt % PAN control fibers, and (c) 11 wt % PAN control fibers. Images are displayed at magnifications of (1) 2k, (2) 5k, and (3) 20k, highlighting surface roughness and polymer chain alignment differences among the fibers.

Figure 4 provides cross-sectional SEM images of control PAN fibers with concentrations of 9 and 11 wt %, with a focus on the fibrous lamellar structures and void distributions within the fibers obtained through tensile testing. Figure 4a–c shows the cross sections of 9 wt % control PAN fibers at different magnifications, where the interface displays a fibrous lamellar structure with prominent voids and discontinuities along the structure. These voids, likely resulting from incomplete polymer chain packing and limited molecular entanglement during the fiber formation process, usually correlate with the observed lower mechanical properties, as voids act as stress concentrators, reducing the fiber’s strength and modulus. In contrast, Figure 4d–f illustrates that the 11 wt % control PAN fibers also exhibit a fibrous lamellar structure but with a denser surface and visibly reduced void content in the cross-sectional images. The increased polymer concentration facilitates better polymer chain alignment and packing during the spinning process, leading to improved molecular interactions and enhanced chain entanglement. This denser structure reduces the likelihood of defects or stress concentration points, contributing to the superior mechanical performance of the 11 wt % fibers, as demonstrated by their higher modulus and strength values (examined in later sections). Moreover, the denser packing likely enhances the crystallinity of the polymer chains, which is supported by literature studies that show improved polymer crystallinity and alignment with higher polymer concentrations in solution.^56–58^ The comparison between the two concentrations highlights the critical role of polymer concentration in influencing fiber morphology. The reduction in voids and improved lamellar density at higher concentrations underscores the importance of optimizing dope formulations to achieve the desired structural and mechanical properties for high-performance applications.^59^

**Figure 4:**
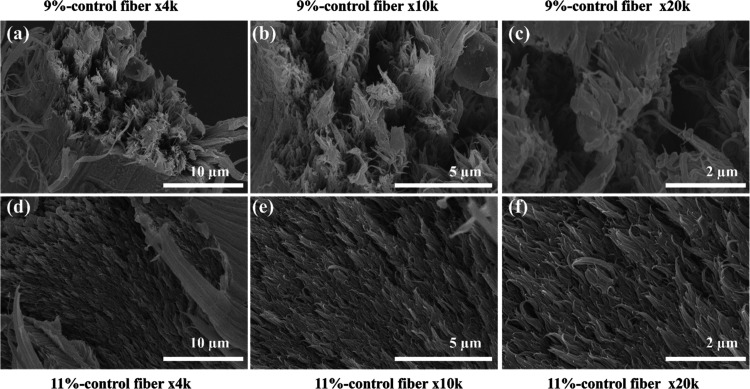
SEM images of the cross-sectional structure of PAN control fibers prepared at different concentrations under three varying magnifications: (a–c) 9 wt % PAN control fibers at 4k, 10k, and 20k magnifications, respectively, and (d–f) 11 wt % PAN control fibers at 4k, 10k, and 20k magnifications, respectively.

### 3.3 CNT Reinforcement of PAN Fibers

Figure 5a provides a schematic representation of the incorporation of carbon nanotubes (CNTs) into the PAN matrix to form PAN-CNT composite fibers. Figure 5b_1_–b_3_ displays the cross sections of PAN-CNT fibers prepared with concentrations of 9, 10, and 11 wt %, respectively. The CNT content in the polymer is 1.8 wt % for a concentration of 9 wt % PAN, 1.6 wt % for 10 wt % PAN, and 1.5 wt % for 11 wt % PAN (i.e., the corresponding volume percentage is 1.2, 1.07, and 0.96 vol %, see the SI). These cross-sectional images were obtained by fracturing the fibers after immersing them in liquid nitrogen. At 9 wt %, the cross section exhibits a fibrous and porous structure, while at 11 wt %, the cross section transitions to a denser and smoother structure, indicating enhanced polymer matrix uniformity with increased concentration. Figure 5c_1_–c_3_ shows the SEM surface morphology of PAN-CNT composite fibers. As the PAN concentration increases to 10 and 11 wt %, the surface becomes progressively smoother, with a noticeable reduction in striations and grooves. These findings highlight the optimal dispersion and alignment of CNTs within the PAN matrix at higher polymer concentrations, contributing to a more uniform fiber structure and potentially enhancing the mechanical properties of the composite fibers.

**Figure 5:**
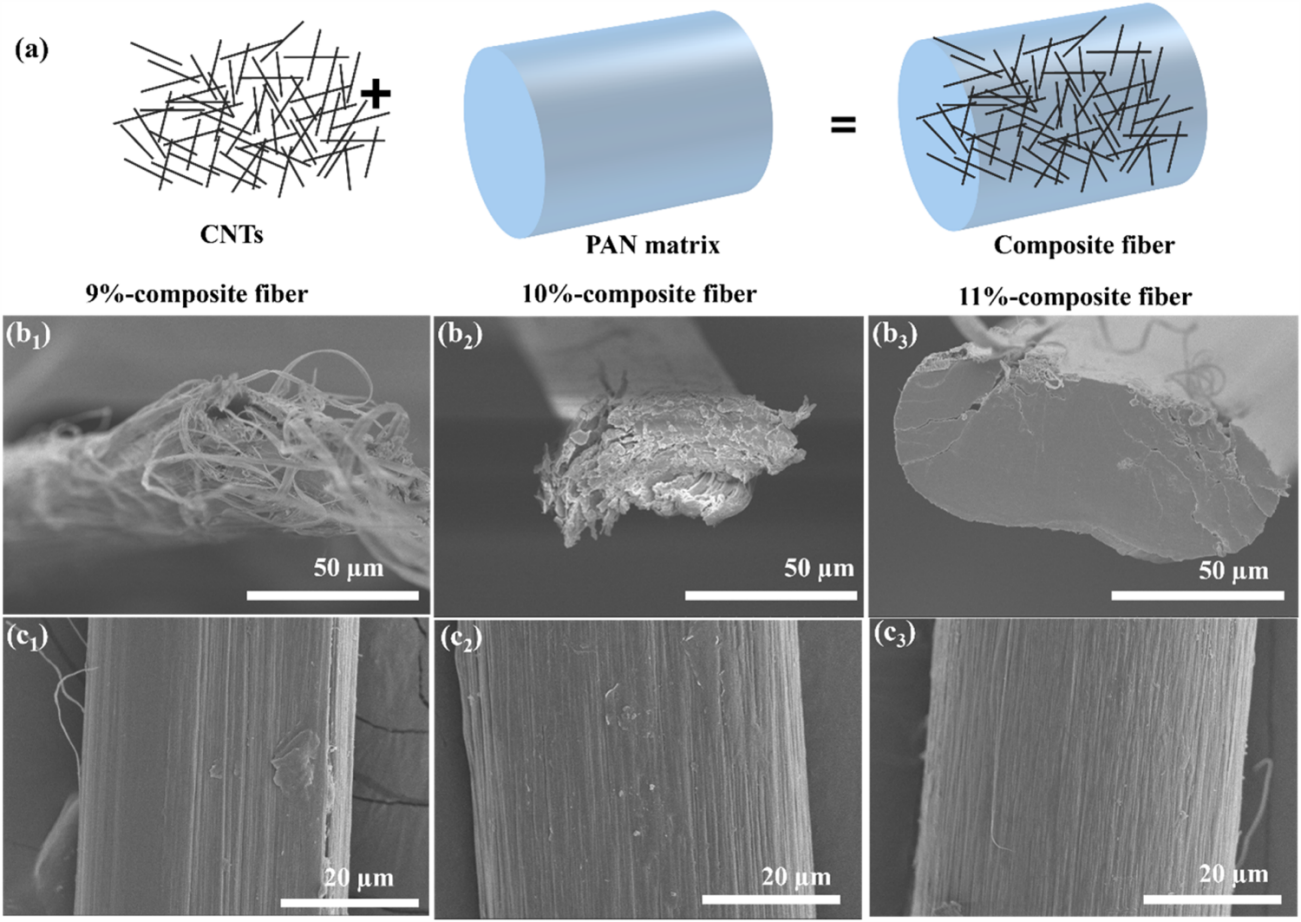
(a) Schematic illustration showing the incorporation of carbon nanotubes (CNTs) into a PAN matrix to form composite fibers. SEM images of the cross-sectional structures of PAN-CNT composite fibers prepared with three different concentrations: (b_1_) 9 wt % PAN-CNT composite fibers, (b_2_) 10 wt % PAN-CNT composite fibers, and (b_3_) 11 wt % PAN-CNT composite fibers. SEM images of the surface: (c_1_) 9 wt % PAN-CNT composite fibers, (c_2_) 10 wt % PAN-CNT composite fibers, and (c_3_) 11 wt % PAN-CNT composite fibers. Scale bars for cross-sectional images: 50 μm; scale bars for surface images: 20 μm.

Figure 6a illustrates the modulus of PAN-CNT composite fibers based on solutions from varying PAN/DMF concentrations, demonstrating an increase from 8.5 GPa at 9 wt % to 9.2 GPa at 10 wt % and reaching 12.8 GPa at 11 wt %. This trend highlights the positive correlation between polymer concentration and fiber modulus. Figure 6b–d compare the modulus of composite fibers with their respective control fibers across different concentrations, clearly showcasing the significant enhancement in mechanical properties achieved through the incorporation of CNTs. The CNTs content in polymer concentrations of 9, 10, and 11 wt % is 1.8, 1.6, and 1.5 wt %, respectively. As mentioned earlier, controlling the dispersion quality of CNTs in polymer/CNT composites is challenging. Higher CNT ratios in the polymer can result in uneven dispersion, leading to poor interfacial interaction between the polymer and CNTs, which in turn decreases the mechanical properties.^23^ A comparison of the Young’s modulus of PAN and PAN-CNT fibers under different conditions across various studies is shown in Table S1.

**Figure 6:**
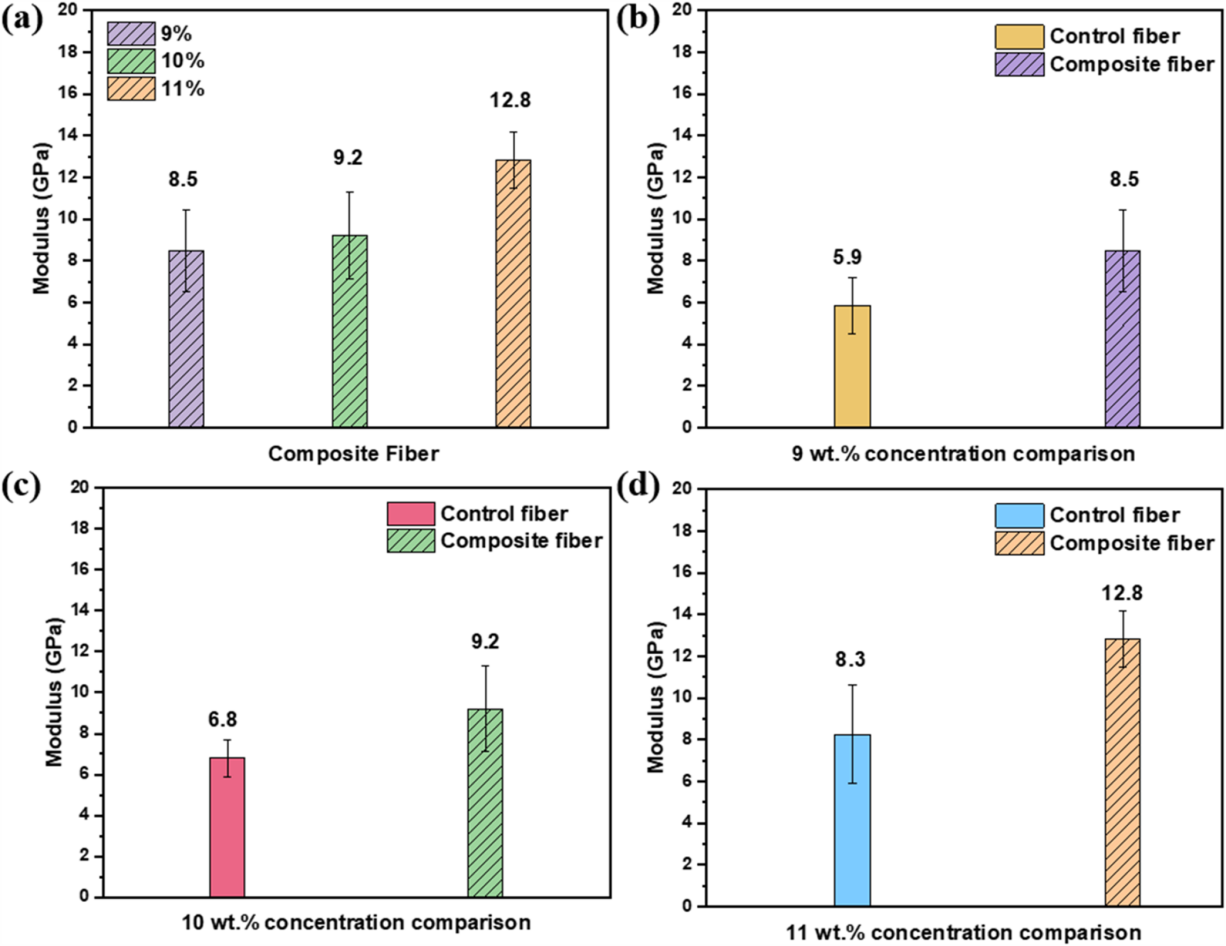
(a) Modulus of PAN-CNT composite fiber at different PAN concentrations of 9, 10, and 11 wt %. Comparison between composite and control fibers based on the PAN/DMF concentration of (b) 9 wt %, (c) 10 wt %, and (d) 11 wt %.

To analyze the CNT reinforcement effects, the mechanical reinforcement of CNTs in PAN-CNT composite fibers was evaluated using the rule-of-mixture principles. Experimental modulus values for the composite fibers were compared to theoretical predictions under the assumption of perfect CNT alignment, which implies full load transfer efficiency. The rule of mixtures was applied, where the composite modulus (*E*_c_) was expressed as a function of the volume fraction of CNTs (*V*_*f*_), matrix modulus (*E*_m_), and CNT modulus (*E*_f_).

By rearranging eq 1, the effective CNT modulus was back-calculated for three polymer concentrations (9, 10, and 11 wt %), with experimentally determined volume fractions of CNTs.

1 $$E_{c}=V_{f}\times E_{f}+\left(1-V_{f}\right)\times E_{m}$$

The predicted CNT modulus values ranged from 222.57, 231.10, and 477.05 GPa, highlighting an increase in reinforcement efficiency at higher PAN concentrations. This approach provides insights into the role of CNTs in enhancing composite mechanical properties under ideal alignment conditions, applicable in fiber material forms, which is also consistent with the literature reports (Table S1 in SI).

To investigate the thermal-mechanical behavior of the fibers, dynamic mechanical analysis (DMA) temperature-ramp tests were performed on 11 wt % PAN-CNT fibers. Additionally, to assess their long-term durability under real-world conditions such as moisture, the tests were conducted with the fibers immersed in water. As shown in Figure S4, both the storage and loss modulus of the PAN-CNT fibers remain stable with increasing temperature. This finding further confirms that the PAN-CNT fibers exhibit strong thermal stability up to 100 °C, even in a moist environment.

### 3.4 CNT Influences on PAN Crystallizations

The crystal structure and molecular orientation of fibers are critical to their mechanical and physical properties. The composition and structure of fibers with different concentrations were analyzed using wide-angle X-ray diffraction (WAXD), while the fibers underwent three stages of hot drawing: 90 °C (stage 1), 120 °C (stage 2), and 150 °C (stage 3). As shown in Table S2, the fiber diameter progressively decreases as the draw ratio increases across different hot-drawing stages (90, 120, and 150 °C). This reduction in diameter is accompanied by a significant improvement in mechanical properties, where Young’s modulus increases from 2.8 GPa (stage 1) to 12.8 GPa (stage 3) and tensile strength improves from 118 to 545 MPa. These findings demonstrate that increasing the draw ratio and processing temperature enhances polymer chain orientation, leading to superior mechanical performance. Additionally, optical microscopy images (Figure S5) provide further evidence of structural refinement with increasing draw ratios, where fiber alignment becomes more uniform, reducing defects and inconsistencies. This highlights the crucial role of draw processing in optimizing fiber properties for high-performance applications. Figure 7 displays the integrated radial WAXD scans with deconvoluted profiles and flat plate photographs of samples for both control fibers and composite fibers based on the 11 wt % PAN/DMF concentration solutions after fibers being treated at various hot-drawing processes. The fibers exhibit two distinct diffraction fringes in the equatorial direction, corresponding to the (100) and (110) crystal planes, respectively, along with four diffraction arcs in the meridional direction, symmetrically distributed along the central axis. As the hot-drawing temperature increases, the diffraction fringes in the equatorial direction become shorter and sharper, indicating higher crystallinity and degree of molecular orientation, which can be seen in both Figure 7a (control fibers) and Figure 7b (composite fibers). The crystallinity was determined by analyzing the integrated scans and the areas of the deconvoluted peaks, as shown in Figure 7c,d. For baseline subtraction, a linear line was drawn between 2θ = 10 and 60°. The crystal size was also determined from the equatorial peak at 2θ–17° using the Scherrer equation.^6,60,61^

**Figure 7:**
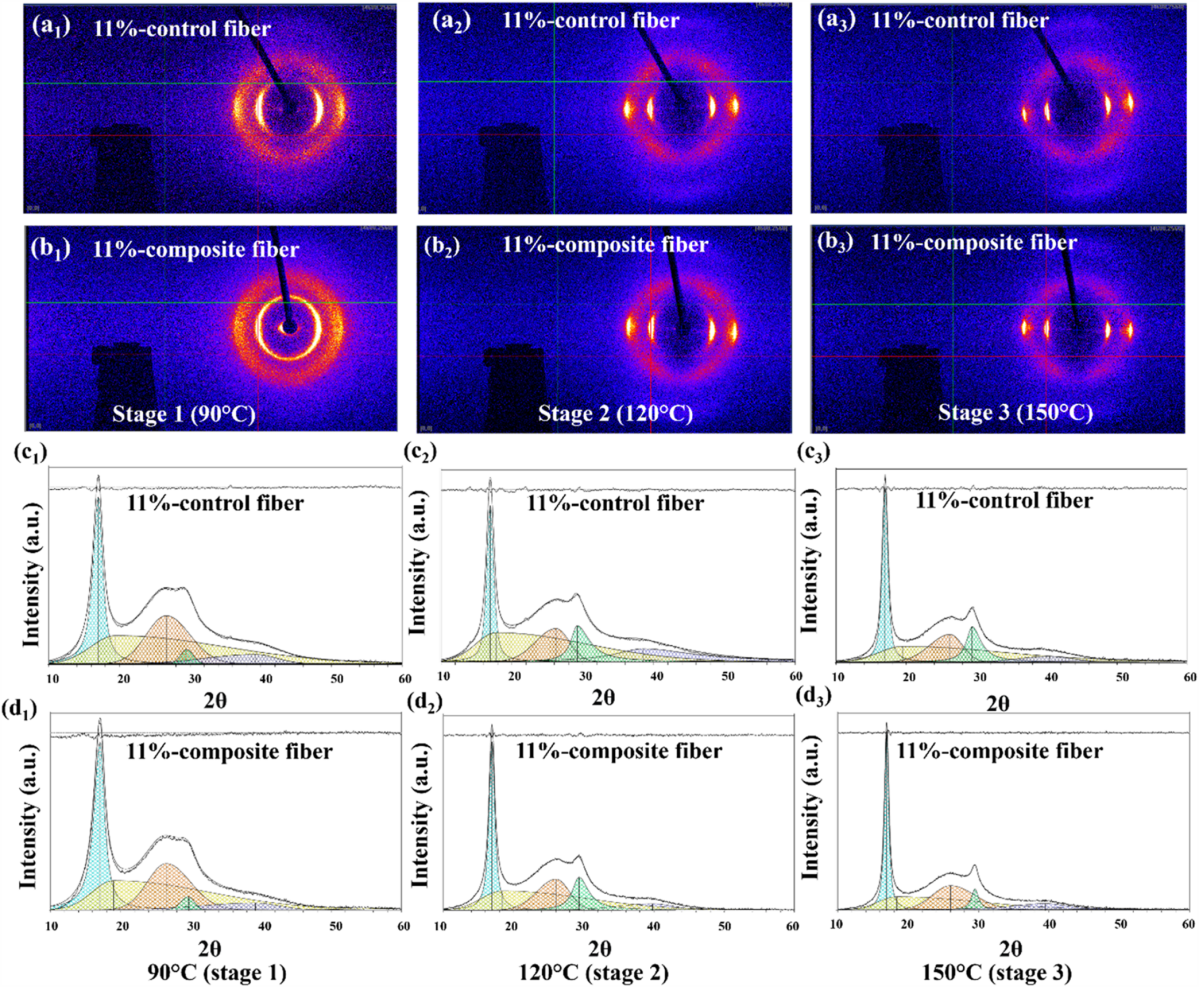
(a–b) Wide-angle X-ray diffraction (WAXD) patterns and (c–d) integrated radial scans with deconvoluted profiles for 11 wt % PAN control and composite fibers at different hot-drawing stages. (a_1_–a_3_) WAXD patterns of 11 wt % control fibers at 90 °C (stage 1), 120 °C (stage 2), and 150 °C (stage 3). (b_1_-b_3_) WAXD patterns of 11 wt % composite fibers at the same stages. (c_1_–c_3_) Integrated radial scans and deconvoluted profiles for 11 wt % control fibers at the respective stages. (d_1_–d_3_) Integrated radial scans and deconvoluted profiles for 11 wt % composite fibers at the corresponding hot-drawing stages of 90, 120, and 150 °C.

The inclusion of CNTs in the PAN matrix significantly enhances the crystallization behavior and crystallite size of the resulting composite fibers, as summarized in Table 1. Composite fibers exhibit consistently higher crystallinity and larger crystallite sizes across all hot-drawing stages compared to control fibers. For instance, at stage 3, the crystallinity of composite fibers reaches 67.6%, compared to 62.4% for control fibers, while the crystallite size increases from 8.2 nm in control fibers to 11.0 nm in composite fibers. This improvement can be attributed to the CNTs serving as nucleation sites, promoting the alignment of PAN molecular chains and facilitating better crystalline packing. The larger crystallites lead to a more rigid and uniform matrix, further enhancing load transfer efficiency and reinforcing the composite Besides, the higher crystallinity results in a more ordered microstructure, which directly contributes to the enhanced mechanical properties, such as higher modulus and strength, and improved thermal stability of the composite fibers. This enhanced molecular orientation in composite fibers contributes to their superior mechanical properties, which align with the observed modulus improvements in Figure 6. The higher alignment and crystallinity of composite fibers indicate their potential for advanced mechanical applications requiring high-performance materials.

**Table 1:** WAXD Results for Control PAN Fibers and Composite PAN-CNTs Fibers at Different Hot-Drawing Stages Based on Results from Figure 7

	control fiber (11 wt %) stage 1	control fiber (11 wt %) stage 2	control fiber (11 wt %) stage 3	composite fiber (11 wt %) stage 1	composite fiber (11 wt %) stage 2	composite fiber (11 wt %) stage 3
draw ratio	3.7	11.0	**22.0**	3.3	11.1	**22.2**
crystallinity (%)	58.2	59.6	**62.4**	60.4	63.6	**67.6**
crystallite size (nm)	4.8	6.7	**8.2**	4.9	8.2	**11.0**

### 3.5 CNT Influences on Composite Thermal Properties

Figure 8a,b illustrates the simultaneous thermal analysis (SDT) curves of PAN control fibers and PAN-CNT composite fibers at various concentrations under a nitrogen atmosphere. A significant acceleration in weight loss is observed between 300 and 400 °C, primarily due to the conversion of hydrogen cyanide and dehydrogenation reactions.^62^ As shown in Figure 8a,b, the thermal stability of PAN fibers with different polymer concentrations exhibits consistent trends, with total weight retention of 32.1 to 33.5% for control PAN fibers and 34.3 to 36.7% for PAN-CNT composite fibers, confirming enhanced residual mass with CNT incorporation. This reinforces the role of CNTs in improving thermal stability, which is critical for carbon fiber production. For PAN-CNT composite fibers, as shown in Figure 8b, the total weight losses are 65.7% for both 9 and 10 wt % fibers, and 63.3% for 11 wt % fibers. Comparing Figure 8a,b, it is evident that PAN-CNT composite fibers exhibit higher residue percentages than PAN control fibers, indicating enhanced resistance to thermal degradation. Differential scanning calorimetry (DSC) thermograms of PAN control fibers and PAN-CNT composite fibers, measured at a heating rate of 10 °C min^–1^, are shown in Figure 8c,d. All fibers exhibit a single exothermic peak, corresponding to the typical cyclization reaction. Figure 8c presents the DSC curves of PAN control fibers and composite fibers with different concentrations under a nitrogen atmosphere. The exothermic peaks are observed at 306.4 °C for the control PAN fibers and 311.3 °C for the composite fibers, indicating that the composite fibers exhibit better regulation of heat release during the preoxidation process compared to the control fibers. Figure 8d shows the DSC curves of the fibers in an air atmosphere. The exothermic peaks occur at 310.1 °C for PAN control fibers and 315.6 °C for composite fibers. These results demonstrate that the incorporation of CNTs enhances the thermal stability of the composite fibers.

**Figure 8:**
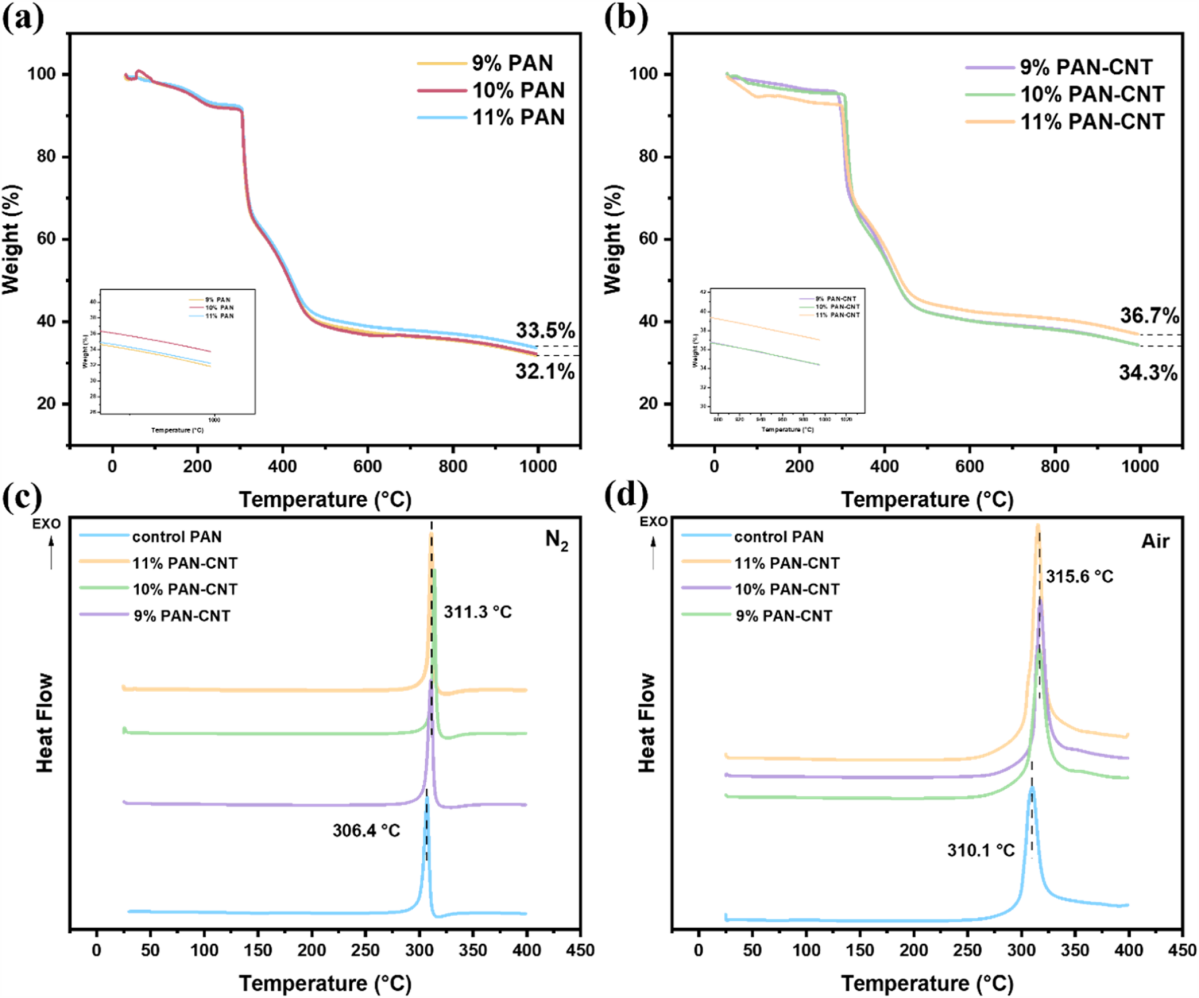
(a) Simultaneous thermal analysis (SDT) plots for PAN control fibers and (b) PAN-CNT composite fibers under an inert nitrogen atmosphere, corresponding to 9, 10, and 11 wt %. Differential scanning calorimetry (DSC) curves comparing PAN control fibers with 9, 10, and 11 wt % PAN-CNT composite fibers under (c) an air atmosphere and under (d) a nitrogen atmosphere.

## 4 Conclusions

This study highlights the enhanced performance of PAN fibers and PAN/CNT composite fibers fabricated at polymer dope concentrations of 9, 10, and 11 wt %. The CNT content in the composite fibers is 1.8 wt % for 9 wt %, 1.6 wt % for 10 wt %, and 1.5 wt % for 11 wt %. Higher polymer concentrations improve mechanical properties, surface smoothness, and reduce voids, with 11 wt % composite fibers achieving exceptional tensile strength (556 MPa) and modulus (13 GPa). Enhanced crystallinity and polymer orientation in composite fibers contribute to their superior properties, while increased polymer concentration improves thermal stability, demonstrating the potential of CNT-based fibers for advanced applications.

## Supplementary Material



## Notes

Additional experimental details are provided, including the calculation of CNT content in the composite polymer, the filtration process, rheological measurements of PAN/DMF solutions, and the effect of DI water on PAN-CNT fibers (covering both morphological and mechanical comparisons); dynamic mechanical analyses on 11 wt % PAN-CNT fibers; fiber comparisons across different hot-drawing stages for 11 wt %; and a discussion of how our work compares with the current state of the art in the literature (PDF)

## ∥

Dr. Minus passed away on Tuesday, August 6, 2024, at the age of 46, all her life focused on researching the fabrication and characterization of advanced high-performance polymer nanocomposites.
