# Supplementary material for: Carbon Nanoparticle Effects on PAN Crystallization for Higher-Performance Composite Fibers
Source: ACS Polym Au. 2025 Apr 9;5(3):270–81. doi: 10.1021/acspolymersau.5c00006 (PMC12163947; doi:10.1021/acspolymersau.5c00006)
Supplement: Supplementary file 1 [file lg5c00006_si_001.pdf]

Supplementary Information
Carbon Nanoparticle Effects on PAN Crystallization for Higher Performance Composite Fibers

Xiao Sun[1], Xiaoli Li[1], Varunkumar Thippanna[2], Conor Doyle[1], Ying Mu[1], Thomas Barrett[1], Lindsay B. Chambers[2], Churan Yu[2], Yiannis Levendis[1], Kenan Song[3,], Marilyn Minus[1,]*[†]*

1 Department of Mechanical and Industrial Engineering, Northeastern University, 360 Huntington Avenue, Boston, Massachusetts, 02115, United States

2 Mechanical Engineering, College of Engineering, University of Georgia, 302 E Campus Rd, Athens, 30602, GA, United States

3 Mechanical Engineering, College of Engineering, University of Georgia (UGA), 302 E. Campus Rd., Athens, 30602, GA, United States

*Corresponding author: kenan.song@uga.edu

[†] Dr. Minus passed away on Tuesday, August 6, 2024, at the age of 46, all her life focus on researching the fabrication and characterization of advanced high-performance polymer nano-composites.

Table of Contents

1. FIBER SPINNING BATCH COMPOSITIONS 3
2. THE FILTRATION PROCESS (A) AND THE PHOTO OF THE OBTAINED INK-LIKE PASTE (B). 4
3. RHEOLOGICAL MEASUREMENTS OF PAN/DMF SOLUTIONS AT (A) 25°C AND (B) 50°C. 5
4. EFFECT OF DI WATER ON 9 WT.% PAN-CNT FIBERS: MORPHOLOGICAL AND MECHANICAL COMPARISON 6
5. THE PHOTOGRAPH OF THE WATER IMMERSION TEST APPARATUS (A) AND DYNAMIC MECHANICAL ANALYSIS RESULTS FOR PAN-CNT FIBERS (B). 7
6. OPTICAL MICROSCOPE IMAGE COMPARING THE CROSS-SECTIONS OF PAN-CNT (11 WT.%) FIBERS (A) AND MECHANICAL ANALYSIS COMPARISON (B). 8
7. OUR WORK AS COMPARED TO THE LITERATURE STATE-OF-THE-ART 9
8. COMPARISON OF VARIOUS PARAMETERS ACROSS DIFFERENT HOT DRAWING STAGES FOR PAN-CNT (11 WT.%) FIBERS 10
9. REFERENCES: 11

1. Fiber Spinning Batch Compositions

The calculation of the different polymer concentrations.

$$\frac{\mathbf{2.8}g(PAN)}{\mathbf{2.8}g(PAN)+\mathbf{0.944}*\mathbf{30ml}(DMF)}=\mathbf{9}\ wt.\%$$

$$\frac{\mathbf{3.15}g(PAN)}{\mathbf{3.15}g(PAN)+\mathbf{0.944}*\mathbf{30ml}(DMF)}=\mathbf{10}\ wt.\%$$

$$\frac{\mathbf{3.5}g(PAN)}{\mathbf{3.5}g(PAN)+\mathbf{0.944}*\mathbf{30ml}(DMF)}=\mathbf{11}\ wt.\%$$

The content of CNTs in composite polymer in weight percentage.

In 9 wt. % $\frac{51.75\ mg\ (CNTs)}{2.8g\ (PAN)*1000+51.75\ mg(CNTs)}=\mathbf{1.8}\ wt.\%$

In 10 wt.% $\frac{51.75\ mg(CNTs)}{3.15g\ (PAN)*1000+51.75\ mg(CNTs)}=\mathbf{1.6}\ wt.\%$

In 11 wt.% $\frac{51.75\ mg(CNTs)}{3.5g\ (PAN)*1000+51.75\ mg(CNTs)}=\mathbf{1.5}\ wt.\%$

The content of CNTs in composite polymer in volume percentage.

In 9 wt. % $\frac{51.75\ mg\ (CNTs)/(1.8*1000\ mg/cm3)}{2.8g\ (PAN)*1000/(1.18*1000\ mg/cm3)+51.75\ mg(CNTs)/(1.8*1000\ mg/cm3)}\sim\mathbf{1.20}\ vol.\%$

In 10 wt.% $\frac{51.75\ mg(CNTs)/(1.8*1000\ mg/cm3)}{3.15g\ (PAN)*1000/(1.18*1000\ mg/cm3)+51.75\ mg(CNTs)/(1.8*1000\ mg/cm3)}\sim\mathbf{1.07}\ vol.\%$

In 11 wt.% $\frac{51.75\ mg(CNTs)/(1.8*1000\ mg/cm3)}{3.5g\ (PAN)*1000/(1.18*1000\ mg/cm3)+51.75\ mg(CNTs)/(1.8*1000\ mg/cm3)}\sim\mathbf{0.96}\ vol.\%$

2. The filtration process (a) and the photo of the obtained ink-like paste (b).

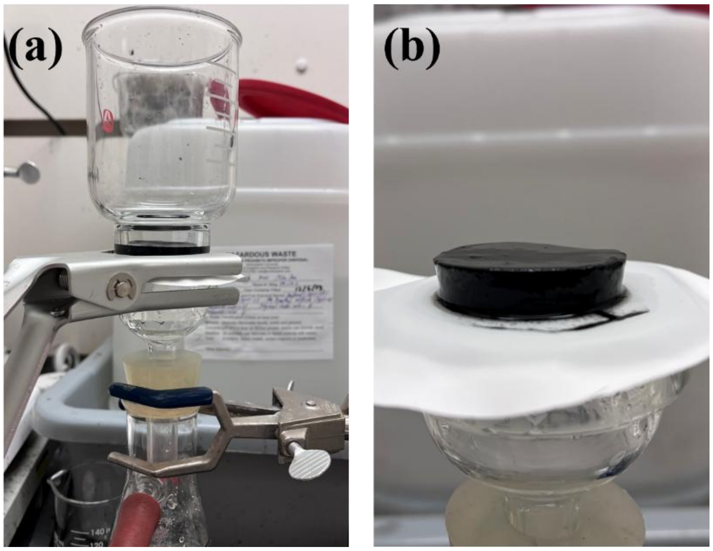

Figure S1. Filtration procedure for the obtaining of carbon inks used in spinning batch

3. Rheological measurements of PAN/DMF solutions at (a) 25°C and (b) 50°C.

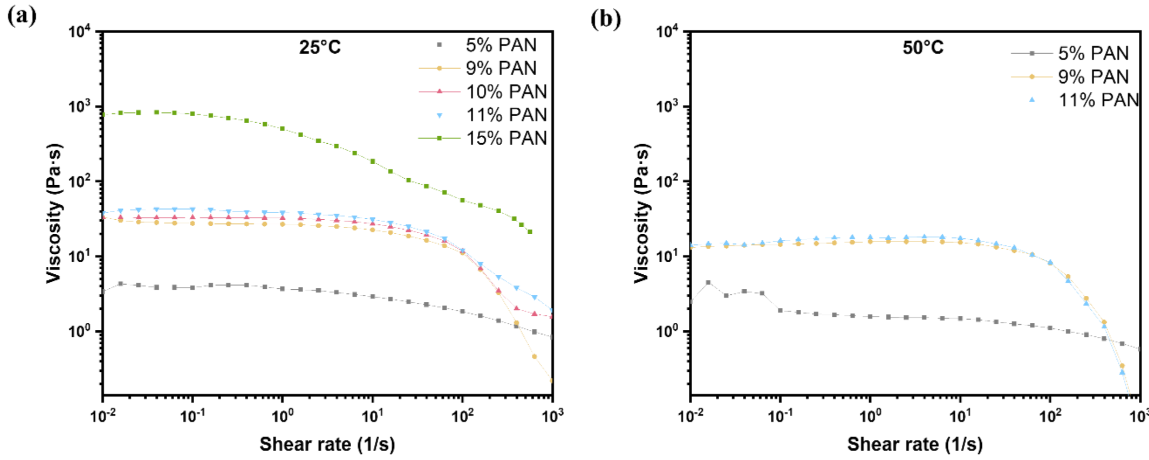

Figure S2. Rheological measurements of 5, 9, 10, 11, and 15 wt.% PAN/DMF solutions at 25°C and 50°C

4. Effect of DI water on 9 wt.% PAN-CNT fibers: morphological and mechanical comparison

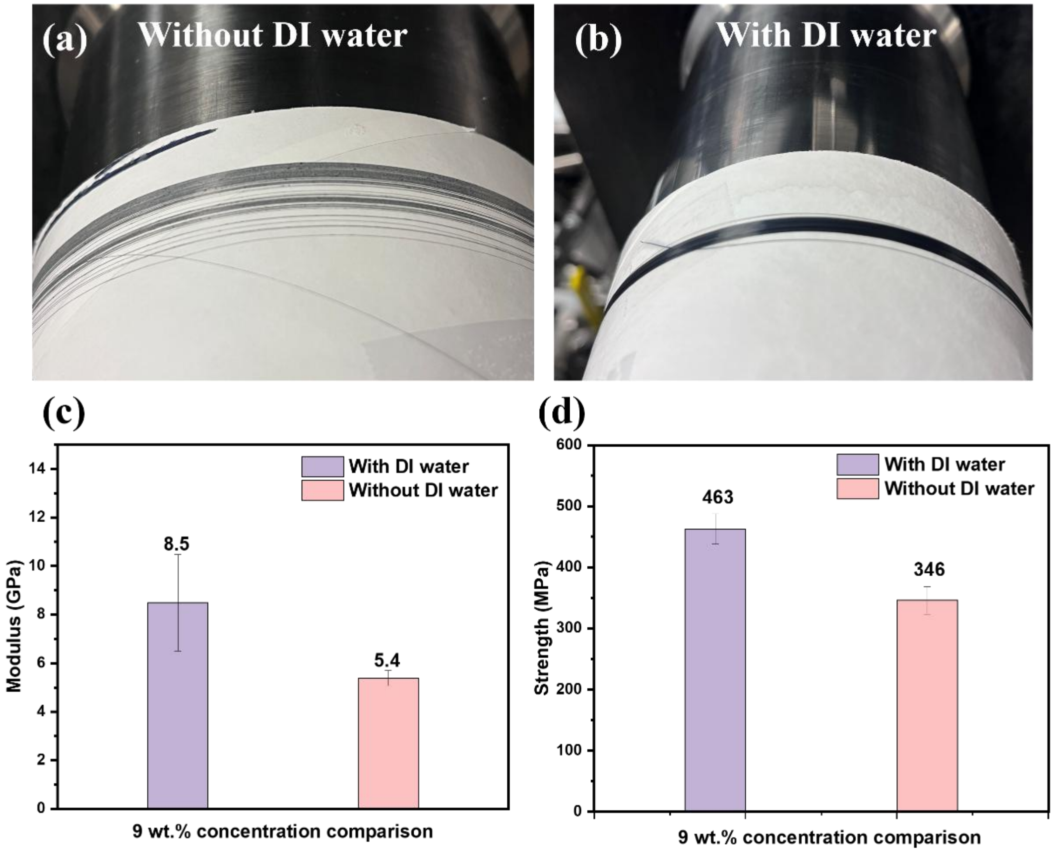

Figure S3. (a) Photo of 9 wt.% PAN-CNT fibers after hot drawing, comparing the traditional method without DI water and our unique method incorporating DI water. (b) Mechanical analysis comparing the modulus and strength of fibers processed with and without DI water.

5. The Photograph of the water immersion test apparatus (a) and dynamic mechanical analysis results for PAN-CNT fibers (b).

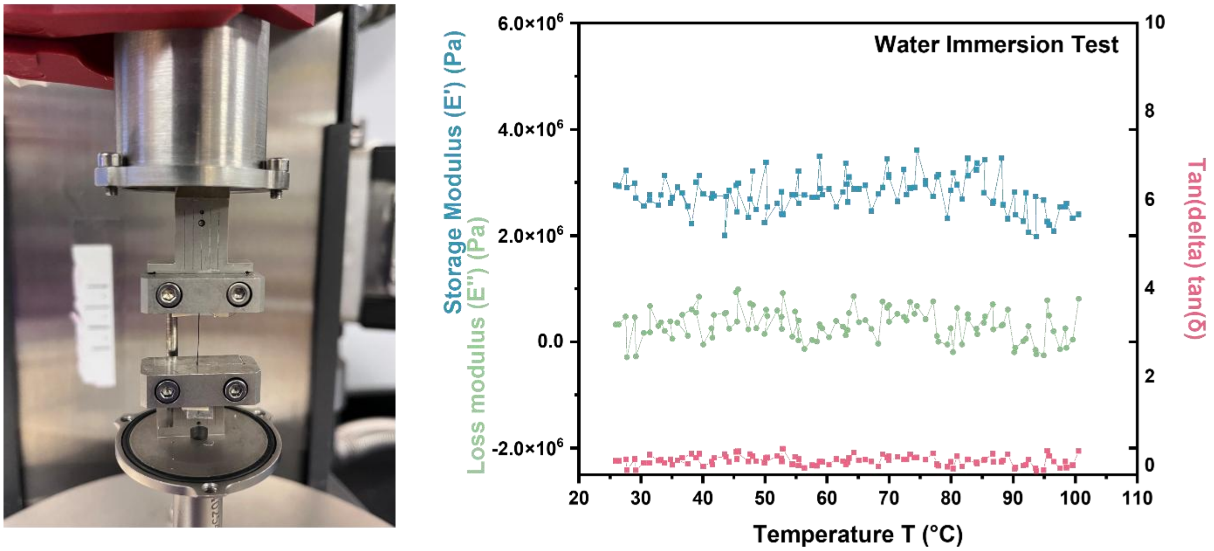

Figure S4. Storage and loss modulus of 11 wt.% PAN-CNT immersed in water during a temperature ramp.

6. Optical microscope image comparing the cross-sections of PAN-CNT (11 wt.%) fibers (a) and mechanical analysis comparison (b).

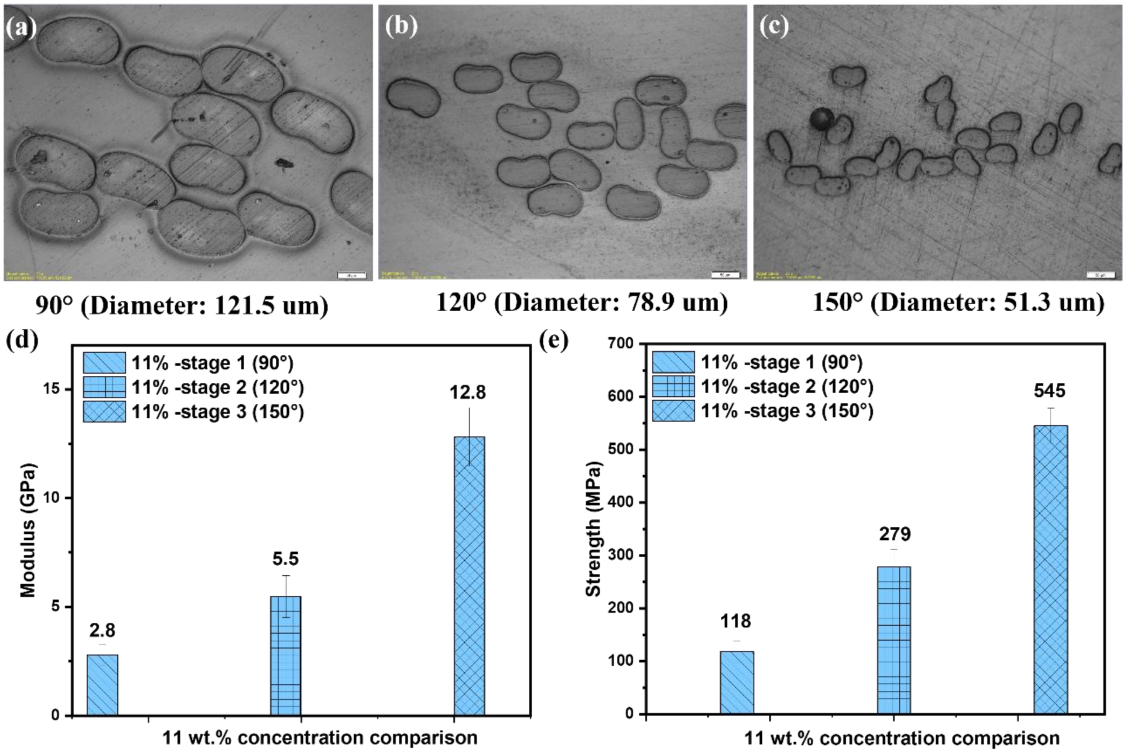

Figure S5. Optical microscope image and corresponding diameter comparison of the cross-sections of PAN-CNT (11 wt.%) fibers (a), along with modulus and strength comparison across three different hot drawing stages (b).

7. Our work as compared to the literature state-of-the-art

Table S1. Comparison of Young's modulus of PAN and PAN-CNT fibers under different conditions across various studies.

Fiber types	Polymer (Mv) (kg/mol)	Solvent	Heat Treated fiber properties		Reference
			CNT content (wt.%)	Youngs Modulus (GPa)	
9 wt.% PAN	230	DMF	0	5.9 ±1.4	This work
9 wt.% PAN-Mixed CNT			1.8	8.5 ±2.0	
10 wt.% PAN			0	6.8 ±0.9	
10 wt.% PAN-Mixed CNT			1.6	9.2 ±2.1	
11 wt.% PAN			0	8.3 ±2.4	
11 wt.% PAN-Mixed CNT			1.5	12.8 ±1.3	
10 wt.% PAN-CNT	247		3.7	5.7 ±0.3	[1]
22 wt.% PAN	100		0	9.7±0.2	[2]
22 wt.% PAN	780	DMSO	0	7.0±0.8	[3]
22 wt.% PAN-MWCNT			0.5	10.5±1.2	
22 wt.% PAN-MWCNT			1	11.4±1.4	
23.5 wt.% PAN-MWCNT	250	DMF	1	10.3±0.3	[4]
23.5 wt.% PAN-SWCNT			1	9.9±0.3	

The table provides a comparative analysis of Young's modulus for PAN and PAN-CNT fibers under various conditions and polymer concentrations. In our work, PAN-CNT fibers with 9 wt.%, 10 wt.%, and 11 wt.% PAN concentrations exhibited a significant increase in Young's modulus compared to their PAN-only counterparts, demonstrating the reinforcing effect of CNTs. For example, at 11 wt.% PAN with 1.5 wt.% CNTs, the modulus reached 12.8 ± 1.3 GPa, a notable improvement over the 8.3 ± 2.4 GPa of PAN-only fibers. This trend aligns with other studies in **Table S1**, with one example showing the inclusion of CNTs (MWCNT or SWCNT) consistently enhances the mechanical performance of fibers, as seen in fibers with 22 wt.% PAN and 0.5 wt.% CNTs achieving a modulus of 10.5 ± 1.2 GPa. The polymer molecular weight (Mv), solvent type, and CNT content play critical roles in determining fiber properties, as higher polymer concentrations or different solvents like DMSO can influence chain alignment and CNT dispersion, further impacting mechanical performance. These results highlight the synergy between optimized PAN concentration and CNT incorporation for high-performance fiber applications.

8. Comparison of various parameters across different hot drawing stages for PAN-CNT (11 wt.%) fibers

Table S2. Comparison of various parameters across three different hot drawing stages, including draw ratio, diameter, modulus, and strength. It is observed that, in addition to the decreasing diameter with increasing draw ratio, both the modulus and strength also improve as the draw ratio increases.

Hot drawing stages	Draw ratio	Diameter (µm)	Modulus (GPa)	Strength (MPa)
90 °C -stage 1	4.3	121.5	2.8	118
120 °C -stage 2	7.2	78.9	5.5	279
150 °C -stage 3	19.3	51.3	12.8	545

9. References:

[1] M. Lu, P.V. Gulgunje, P.J. Arias-Monje, J. Luo, J. Ramachandran, Y. Sahoo, S. Agarwal, S. Kumar, Structure, properties, and applications of polyacrylonitrile/carbon nanotube (CNT) fibers at low CNT loading, Polymer Engineering & Science, 60 (2020) 2143-2151.

[2] R. Jain, H.G. Chae, S. Kumar, Polyacrylonitrile/carbon nanofiber nanocomposite fibers, Composites science and technology, 88 (2013) 134-141.

[3] J. Zhang, Y. Zhang, D. Zhang, J. Zhao, Dry-jet wet-spun PAN/MWCNT composite fibers with homogeneous structure and circular cross-section, Journal of Applied Polymer Science, 125 (2012) E58-E66.

[4] T. Mikolajczyk, G. Szparaga, M. Bogun, A. Fraczek-Szczypta, S. Blazewicz, Effect of spinning conditions on the mechanical properties of polyacrylonitrile fibers modified with carbon nanotubes, Journal of applied polymer science, 115 (2010) 3628-3635.
